# Supplementary material for: Natural Oscillatory Frequency Slowing in the Premotor Cortex of Early-Course Schizophrenia Patients: A TMS-EEG Study
Source: Brain Sci. 2023 Mar 24;13(4):534. doi: 10.3390/brainsci13040534 (PMC10136843; doi:10.3390/brainsci13040534)

Supplementary material

Table S1

Summary of TMS-EEG measures. Means and SDs of Natural Frequency, Global and Local Mean Field Powers (GMFP and LMFP), and Event-Related Spectral Perturbation (ERSP) values (calculated both in a local ROI and across all channels) are reported for both groups, alongside the p values after Wilcoxon rank-sum test. Alpha: 8-12 Hz; beta: 13-30 Hz; gamma: 30-45 Hz. HC: healthy controls; ECSCZ: early-course schizophrenia patients.

	HC	ECSCZ	p val
Natural Frequency	29.15 ± 6.59	23.27 ± 4.27	0.0186
GMFP	0.77 ± 0.69	0.61 ± 0.43	0.6375
LMFP	0.92 ± 0.91	0.56 ± 0.42	0.2662
ERSP (all freq, global)	0.53 ± 0.55	0.43 ± 0.30	0.8950
ERSP (all freq, local)	1.11 ± 0.96	0.77 ± 0.71	0.1935
ERSP (alpha global)	0.57 ± 1.02	0.13 ± 0.30	0.1365
ERSP (alpha local)	1.07 ± 1.62	0.23 ± 0.57	0.1177
ERSP (beta global)	0.58 ± 0.60	0.50 ± 0.40	0.9849
ERSP (beta local)	1.23 ± 1.05	0.95 ± 0.92	0.3757
ERSP (gamma global)	0.45 ± 0.36	0.43 ± 0.26	0.5591
ERSP (gamma local)	0.92 ± 0.60	0.70 ± 0.52	0.2827

Figure S1

Global Mean Field Power – group comparison. Figure shows grand averages of the Global Mean Field Power (GMFP, top) for healthy controls (blue) and early-course schizophrenia patients (red). Bottom panel shows group comparisons for 50ms time bins. No significant differences were found for any time bin (Wilcoxon rank-sum tests, α<0.05, uncorrected).

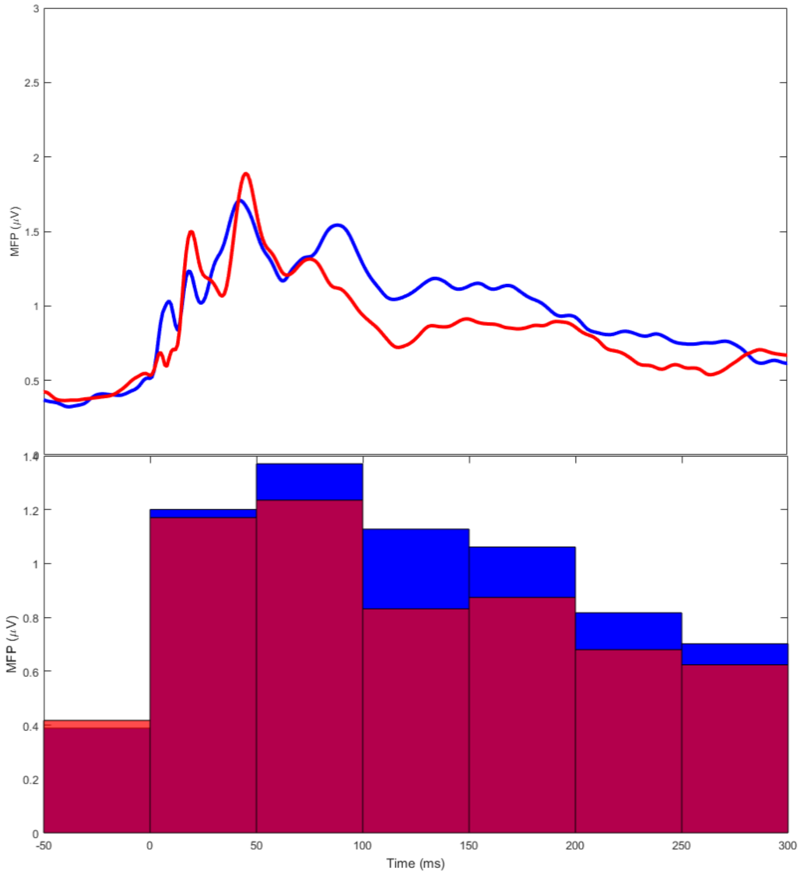

Figure S2

Local Mean Field Power – group comparison. Same as Figure S1 but limited to a cluster of electrodes close to the stimulation site (i.e., Local MFP, LMFP). No significant differences were found for any time bin (Wilcoxon rank-sum tests, α<0.05, uncorrected).

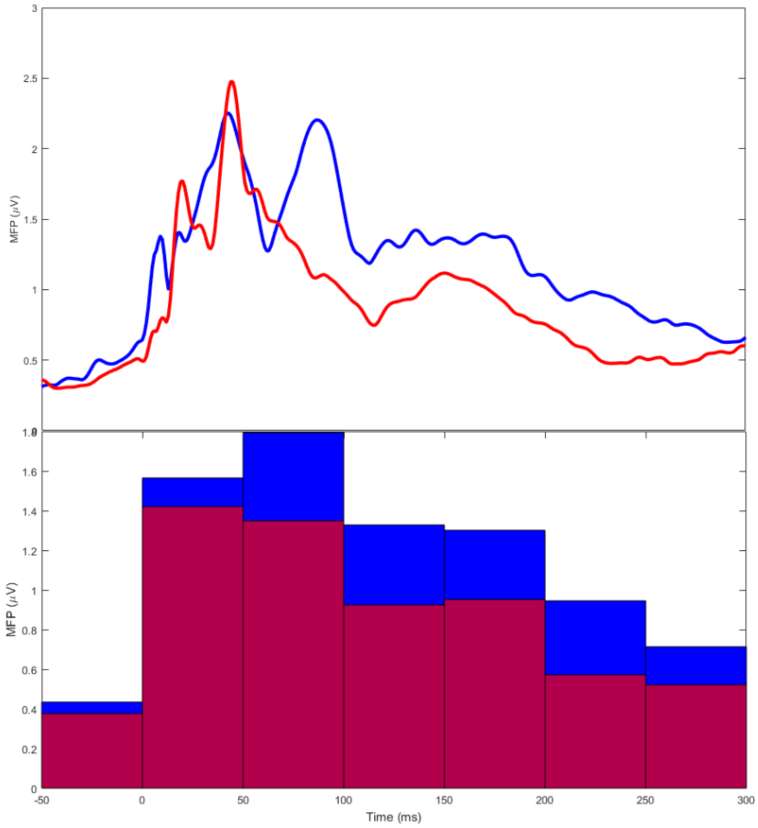

Supplement: Supplementary file 1 [file brainsci-13-00534-s001.zip › brainsci-2249599-supplementary.pdf]
